# Equity on the Mother and Baby Unit: An Audit of Detention Rates and Length of Stay According to Ethnicity and English Language Ability

**DOI:** 10.1192/bjo.2024.627

**Published:** 2024-08-01

**Authors:** Najla Eiman, Morwenna Senior, Sarah Jones

**Affiliations:** 1 Cheshire and Wirral NHS Foundation Trust, Chester, United Kingdom; 2 Greater Manchester Mental Health NHS Foundation Trust, Manchester, United Kingdom

## Abstract

**Aims:**

A large body of evidence suggests that experiences of, and access to mental healthcare in England varies according to ethnicity. Inequitable use of restrictive interventions is of particular concern, including within perinatal services: a national-level study of inpatients on Mother and Baby Units (MBU) in 2017 found that 28% of white patients were detained under the mental health act (MHA), compared with 61.5% of Black African, and 66 – 77% of Asian mothers. We carried out an audit with the aim of examining detention rates, length of stay, and time to first section 17 leave on an MBU in South Manchester according to ethnicity and English language ability, to compare with national averages.

**Methods:**

We identified all patients discharged from Andersen Ward (an MBU) between March 2022 and March 2023. Using electronic medical records we extracted information on: ethnicity, language spoken (English vs other), mental health act status (detained under Section 2/3 vs informal), duration of admission, date of detention, date of first Section 17 leave. We calculated the percentage of patients who were detained according to ethnicity (White British, Mixed/other, Asian, Black), and the odds of detention according to ethnicity. Statistical significance was assessed using chi-squared testing. We also compared average length of stay and time to first section 17 leave by ethnicity.

**Results:**

74 patients had been discharged from the MBU within the audit period. 88% of Black inpatients were admitted under the Mental Health act, compared with 72.7% of Asian mothers, 33.3% of Mixed ethnicity or other ethnicities and 28.3% of white mothers. Differences in detention rates according to ethnicity were statistically significant. Of 11 mothers documented as having a language other than English as their primary language, all had been detained. Length of admission and days to first section 17 leave were not significantly different between ethnicities.

**Conclusion:**

Many factors may contribute to the observed higher detention rates among non-White patients: language barriers and a lack of intercultural competence could lead to risk-averse decision-making during MHA assessments, and different help-seeking patterns might mean White mothers seek help earlier, or for less severe mental health problems. Recommendations include expanding access to high-quality interpreters; investigating factors underlying MHA decision-making through qualitative research; and improving cultural competence among section 12 approved clinicians by incorporating feedback from ethnic minority patients into training and refresher courses.

